# Absence of phlebotominae sandflies (Diptera: Psychodidae) and allochthonous canine leishmaniasis cases in the Santa Catarina Plateau, Brazil

**DOI:** 10.1590/S1984-29612025012

**Published:** 2025-04-07

**Authors:** Faiane Reila Sousa Centenaro Duarte, Geanice Ledo, Felipe Rieth de Lima, Mayckon Antônio Cardoso Padilha, Márcio Rodrigues da Silva, Manuela Steil Korb, Anderson Barbosa de Moura, Andreas Lazaros Chryssafidis

**Affiliations:** 1 Laboratório de Parasitologia e Doenças Parasitárias – LAPAR, Departamento de Medicina Veterinária, Centro de Ciências Agroveterinárias – CAV, Universidade do Estado de Santa Catarina – UDES, Lages, SC, Brasil; 2 Centro de Controle de Zoonoses – CCZ, Prefeitura Municipal de Lages, Lages, SC, Brasil

**Keywords:** Entomofauna, zoonosis, one health, *Leishmania* spp, Entomofauna, zoonose, saúde única, *Leishmania* spp

## Abstract

Leishmaniasis is a parasitic disease caused by *Leishmania* spp., transmitted to hosts through the bite of female phlebotomine sandflies, with domestic dogs serving as reservoirs for the disease. Understanding the entomofauna is crucial for effective control of vector-borne diseases, such as leishmaniasis, because various environmental and climatic factors can influence sandfly presence and distribution. This study aimed to conduct a quantitative and qualitative survey of the entomofauna in urban and peri-urban areas of Lages, Santa Catarina, Brazil, and to analyze documented cases of canine leishmaniasis in the city, in response to the rising number of non-autochthonous cases in dogs. The lack of prior studies on this fauna in the municipality raises concerns for public health services. Nine areas were monitored over the course of one year, and clinical and epidemiological records of canine leishmaniasis in the city were analyzed. A total of 10,638 insects were collected, with no phlebotomine sandflies identified. All evaluated cases of canine leishmaniasis were classified as non-autochthonous based on the movement history of these animals and the absence of the disease vector in the municipality. This information may guide further control and prevention measures for leishmaniasis in the region, aligned with a One Health perspective.

## Resumo

Leishmaniose é uma doença parasitária causada por *Leishmania* spp., transmitida aos seus hospedeiros por meio da picada de fêmeas de flebotomíneos, sendo os cães domésticos reservatórios da doença. O conhecimento da entomofauna é crucial para estratégias de controle efetivas de doenças transmitidas por vetores, especialmente leishmaniose, já que vários fatores ambientais e climáticos podem influenciar a presença e distribuição dos flebotomíneos. Este trabalho teve como objetivo conduzir um levantamento qualitativo e quantitativo da entomofauna, com ênfase em flebotomíneos, em regiões urbanas e periurbanas do município de Lages, Santa Catarina, Brasil, e analisar casos registrados de leishmaniose canina na cidade, em resposta ao número crescente de casos não autóctones em cães. A falta de estudos prévios sobre a entomofauna da cidade gera preocupações para serviços de saúde pública. Nove áreas do município foram monitoradas durante um ano, e dados clínicos e epidemiológicos de casos registrados de leishmaniose canina na cidade avaliados. Um total de 10.638 insetos foram coletados, sem identificação de flebotomíneos. Todos os casos de leishmaniose canina foram confirmados como não-autóctones, baseados no histórico de movimento desses animais e na ausência do vetor da doença no município. Essas informações podem guiar medidas preventivas e de controle da leishmaniose na região, alinhadas a uma perspectiva de Saúde Única.

## Introduction

The *Leishmania* genus includes the etiological agents responsible for leishmaniasis, zoonotic diseases transmitted to mammals through the bite of female phlebotomines, popularly known as sandflies (Diptera Order; Psychodidae Family; Subfamily Phlebotominae) (Costa et al., 2019).

Leishmaniases are traditionally categorized into two clinical forms: Visceral Leishmaniasis (VL), which predominantly affects the internal organs, and Cutaneous Leishmaniasis (CL), which primarily affects the skin and its appendages (Dantas-Torres, 2009, 2024). *Leishmania infantum* (syn. *Leishmania chagasi*) is the causative agent of VL in South America, mainly transmitted by *Lutzomyia longipalpis* in Brazil, with dogs being considered its principal urban reservoir (Dantas-Torres, 2009, 2024).

In Brazil, the etiological agents of CL include *Leishmania* (*Viannia*) *braziliensis*, *Leishmania* (*Viannia*) *guyanensis*, and *Leishmania* (*Leishmania*) *amazonensis* (Dantas-Torres, 2024). These species are transmitted by various sandflies from the Psychodidae family and have a wide array of animal reservoirs (Dantas-Torres, 2024; PAHO, 2023).

Canine leishmaniasis generates a broad range of clinical signs, including onychogryphosis, weight loss, skin lesions with alopecia, neurological symptoms, splenomegaly, lymphadenopathies, and ophthalmopathies. However, most dogs remain asymptomatic, highlighting their role as disease reservoirs (PAHO, 2023; Schäfer et al., 2022). Leishmaniases, including human and canine forms, are vector-borne diseases. Understanding the distribution of potential vectors, especially sandflies, in urban and peri-urban areas is crucial for public health, especially within a one-health approach influenced by changes in vector habitats (Dias et al., 2007). Epidemiological investigations into the occurrence and sandfly distribution in Brazil helps control measures for leishmaniasis in both endemic and non-endemic regions. This is crucial as leishmaniasis is a neglected tropical disease with a wide geographic distribution in the country (Costa et al., 2019).

Entomofauna surveys in the Serrana mesoregion of Santa Catarina, Brazil are scarce. This study aimed to conduct a qualitative and quantitative survey of phlebotomine sandflies in the urban and peri-urban areas of the municipality of Lages, Santa Catarina, Brazil, and to investigate the epidemiology of monitored canine leishmaniasis cases in the city, prompted by the rise in non-autochthonous cases, potentially linked with the presence of sandflies in the city.

## Material and Methods

### Characterization of the area of study

The municipality of Lages (27°48’ S, 50°20’ W) covers 2.637.660 km^2^ and has 164.971 habitants (IBGE, 2022). Located in the Serrana Mesoregion, at the Santa Catarina Plateau at an average altitude of 961 m above sea level. According to the Köppen-Gelger scale, the municipality has a temperate oceanic climate (*Cfb*), configuring a humid and temperate climate characterized by mild summers and well-defined seasons, and a distinct dry period (Pandolfo et al., 2002; Ricken et al., 2018; Vieira & Dortzbach, 2017). The average annual temperature ranges from 13.8 to 15.8 °C, with 78-82% humidity and 1300-1700 mm of annual rainfall (Pandolfo et al., 2002; Ricken et al., 2018).

Colder temperatures are typically recorded between May and September, with the coldest, sometimes negative, temperatures occurring from June to August (Vieira & Dortzbach, 2017). The region’s vegetation is classified as Mata Atlântica with mixed rainforest, also known as Araucária forests due to the considerable presence of the Paraná-pine (*Araucaria angustifolia*), combined with highland grasslands (Vieira & Dortzbach, 2017). The peri-urban areas feature small properties that rear livestock such as cattle.

### Entomofauna survey

**Figure 1:**
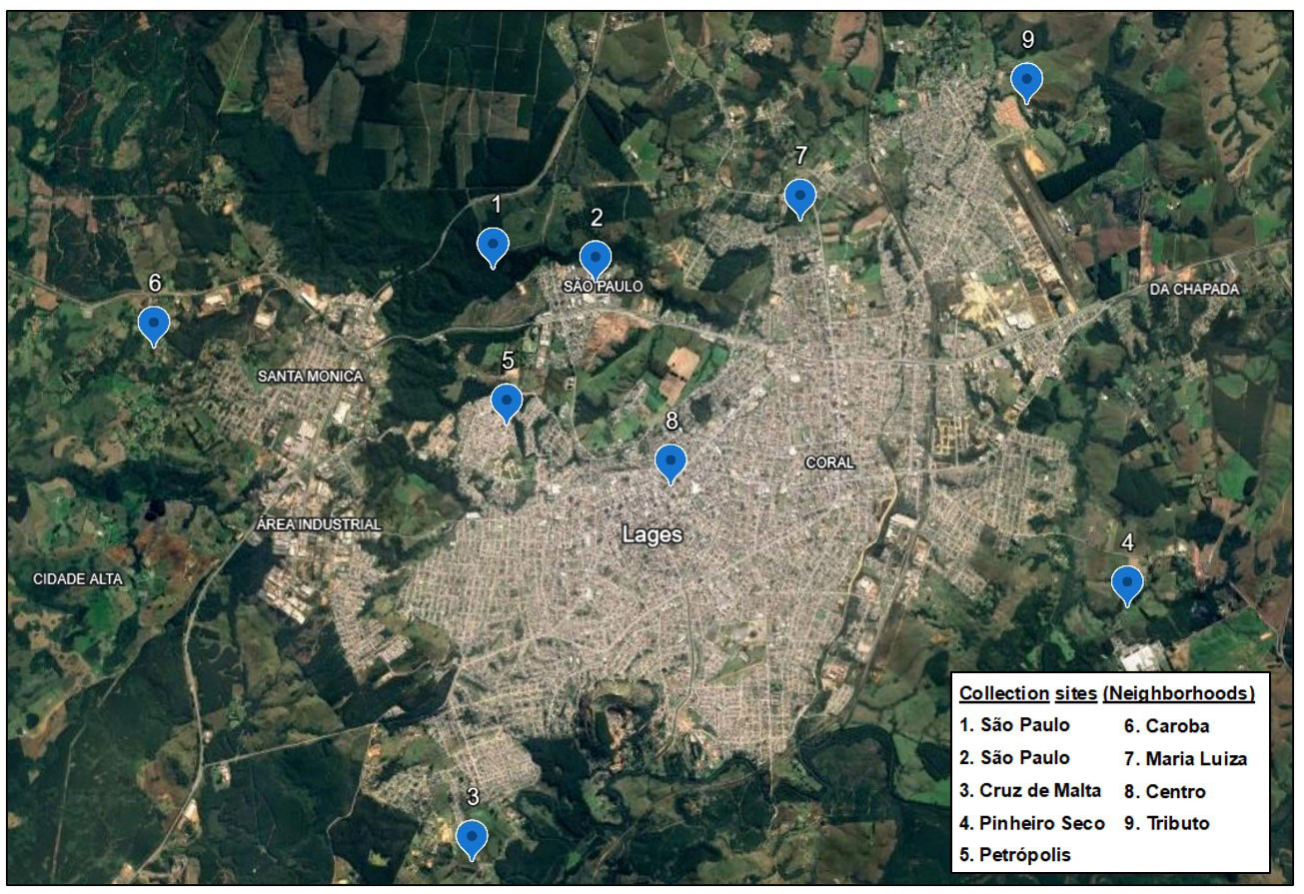
Spatial distribution of insect collection sites at the municipality of Lages. Source: Google Earth (2024).

Insect collection was conducted from August 2019 to July 2020 at nine distinct locations within Lages (Figure 1) using CDC light traps. The locations (1-9) and corresponding neighborhoods were: 1 and 2 (São Paulo), 3 (Cruz de Malta), 4 (Pinheiro Seco), 5 (Petrópolis), 6 (Caroba), 7 (Maria Luiza), 8 (Centro), and 9 (Tributo). Except for location 8 (Centro), all were situated on the outskirts of the municipality near forest boundaries. Location 1-7 were selected for their proximity to forested areas and cattle-rearing farms to enhance the likelihood of capturing sandflies, while location 8 was chosen due to its closeness to the residences of the dogs epidemiologically evaluated for visceral canine leishmaniasis within the city.

A trap was deployed at each of the nine locations for three consecutive nights, and this process was repeated three times for each location, resulting in nine samples collected per location each month. The traps were positioned at different locations at dusk, 1.5 meters above the ground, and left overnight (Silva et al., 2019). The sampling effort was approximately 324 h per trap, totalizing 3.384 hours overall.

Captured insects were transported to the laboratory at the Center for Zoonosis Control in Lages, where they were frozen, stored, and identified based on their location and date of collection. All specimens were cleaned using tweezers to remove major dirt and debris, followed by identification and counting using dichotomous keys (Forattini, 1962).

### Follow-up on local cases of canine leishmaniasis

Between 2018 and 2020, the Center for Zoonosis Control in the municipality of Lages monitored six documented cases of canine leishmaniasis. To investigate their infection and potential correlation with the presence of sandflies in the area, the clinical records of these animals were analyzed based on epidemiological factors, including their place of residence during diagnosis and treatment, vaccination history, and movement patterns.

### Statistical analysis

In the entomofauna survey, with an emphasis on sandflies, the relative frequency of each order was calculated as a percentage of specimens in each order relative to the total number of specimens collected, using the Formula 1:


$$F\;=\;\left(Ni/N\right)\;*\;100$$ (1)

where *F* represents the relative frequency, *Ni* denotes the total number of specimens of the specific Order and *N* is the total number of specimens collected across all orders (Botelho et al., 1976).

Constancy was calculated based on the percentage of occurrence of the orders, using the Formula 2:


$$C\;=\;\left(Ci/Nc\right)\;*\;100$$ (2)

where *C* represents the constancy percentage, *Ci* is the number of collections containing the Order and *Nc* signifies the total number of collections.

Orders were categorized based on the percentage of collections as constant (present in more than 50% of collections), accessory (present in 25-50% of collections), or accidental (present in less than 25% of collections) (Botelho et al., 1976).

## Results

### Entomofauna survey and analysis

Across all nine locations, 282 collections were conducted, resulting in 10,638 specimens. The insects were classified into five Orders: Diptera, Lepidoptera, Hymenoptera, Coleoptera and Orthoptera. Diptera was the most prevalent order, with a relative frequency of 81.86%, followed by Lepidoptera (17.35%), Hymenoptera (0.65%), Coleoptera (0.08%), and Orthoptera (0.05%).

Diptera and Lepidoptera were classified as constant, with Diptera found in 100% of collections and Lepidoptera in 93.26%. The remaining orders were classified as accidental: Hymenoptera was found in 6,02%, Coleoptera in 1,7% and Orthoptera in 1,06%. Within Diptera, specimens from the Culicidae and Psychodidae families (subfamily Psychodinae) were identified. The main Culicidae species found was *Culex quinquefasciatus*. However, no sandflies were found at the collection sites.

### Confirmation of non-autochthonous cases of canine leishmaniasis

Based on the clinical records of dogs positive for VL (Table 1), all animals had previously moved from or originated in a recognized endemic area of the disease. These animals were diagnosed using Enzyme-Linked Immunosorbent Assay (ELISA). They were receiving treatment in accordance with the current Brazilian legislation (Brasil, 2016) while residing in Lages. No record instances of further animal movements were observed during the monitoring period.

**Table 1:** Epidemiological data concerning monitored canine positive cases of visceral leishmaniasis at the municipality of Lages.

**Dog Identification**	**History of movement to endemic area (Country/State/Municipality)**	**Year of diagnosis**	**End of monitoring**	**Vaccination**	**Neighborhood of residence**
**01**	Brazil/Paraíba/João Pessoa	2018	2021	Yes	Centro
**02**	Brazil/Paraíba/João Pessoa	2019	2021	Yes	Centro
**03**	Brazil/Paraíba/João Pessoa	2019	2019	Yes	Centro
**04**	Brazil/Tocantins/Palmas	2019	2020	No	Centro
**05**	Brazil/Federal District/ Brasília	2020	2022	Yes	São Cristóvão
**06**	Spain	2020	2022	No	Vila Mariza

Four of the six animals were housed near the eighth location (Centro) of the entomofauna survey with three of them belonging to the same owner. Of the remaining two animals, one lived near the eighth collection point (São Cristovão) and the other between the fourth and ninth collections (Vila Mariza and Tributo). Additionally, four out of the six animals had been vaccinated with Leish-Tec® (Hertape Calier Saúde Animal) against leishmaniasis, but there was no information on their serological status before or after vaccination.

## Discussion

The absence of sandflies in the survey aligns with the epidemiological data on canine leishmaniasis cases, reinforcing their non-autochthonous nature and emphasizing the necessity of vector presence for transmission. The body temperature of insects is closely linked to the environmental temperature, affecting their physiological processes (Régnière et al., 2012). Various factors, including rainfall, air temperature, wind speed, relative humidity, soil properties (moisture, pH, organic carbon), altitude, forest litter, human activity, and broader climatic phenomena, such as climate change, influence sandfly population dynamics, oviposition, larval survival, and development (Cabaniel et al., 2005; Chowdhury et al., 2016; Nieves et al., 2015; Rangel & Vilela, 2008; Rutledge & Ellenwood, 1975).

Sandfly habitats are characterized by relatively stable temperature and humidity conditions, which are crucial for their persistence, owing to their sensitivity to desiccation (Dias et al., 2007). Cold, dry periods, and excessive rainfall with soil inundation, can reduce sandfly populations, possibly because of unfavorable climatic conditions for the development of immature stages (Dias et al., 2007; Rutledge & Ellenwood, 1975). Rainfall patterns, humidity levels, and other physiographical features are significant factors influencing sandfly presence and seasonality, particularly regarding breeding cycles and soil breeding sites (Rutledge & Ellenwood, 1975).

Sandflies are commonly found in warmer temperatures, moderate rainfall, and high humidity, especially in forested areas due to their ecology (Costa et al., 2019; Dias et al., 2007; Rutledge & Ellenwood, 1975). Most of the collection sites in this study were located on the outskirts of the municipality, near transitional or fully forested areas, but did not affect sandfly occurrence. The city’s precipitation pattern features significant year-round rainfall. Even during dry periods, some rainfall was observed, with an average annual rainfall of 1024.9 mm during the study compared to an expected annual range of 1300–1700 mm (Pandolfo et al., 2002).

According to data from the Lages experimental station provided by the Agricultural Research and Rural Extension Company of Santa Catarina, Santa Catarina Environmental Resources, and Hydrometeorology Information Center (EPAGRI-CIRAM), the study period documented a range of climatic conditions. The coldest recorded temperature was -1.4 °C in August 2019, while the highest temperature was 33.6 °C in December 2019. The average annual temperature was 15.7 °C. Daily rainfall varied significantly, with a minimum of 27.8 mm in January 2020 and a maximum of 178 mm recorded in November 2019.

The colder temperatures typically recorded in Lages, particularly during late autumn and winter, often reaching negative values, are usually accompanied by high humidity and substantial rainfall ˗˗ climatic conditions shared with other municipalities in the Serrana Mesoregion. Although these rainfall patterns and humidity levels might seem adequate for sandfly development, the city’s annual average temperature, along with fluctuations throughout the year, suggests that Lages currently lacks the edaphoclimatic conditions necessary for phlebotomine colonization and sustained presence. This may help explain the absence of autochthonous cases of canine leishmaniasis in the municipality.

Globally, an estimated 0.2 to 0.4 million cases of human VL occur annually (Alvar et al., 2012), with a fatality rate of 95% if untreated (Torres de Sousa et al., 2019). According to the Pan-American Health Organization, between 2001 and 2020, 67,845 human VL cases were reported across 13 countries, with 80% of the cases occurring in Brazil, Ethiopia, Eritrea, India, Kenya, and Sudan. Brazil accounted for 96% of these cases, with an annual average of 3,268 cases (PAHO, 2023).

In Brazil, VL epidemics have been observed in major urban centers since the 1980s, marking the beginning of the disease’s urbanization (Jeronimo et al., 1994). Urbanization, along with climate and environmental changes, has expanded sandfly habitats that were traditionally rural (Dias et al., 2007; Schäfer et al., 2022). This expansion has led to the occurrence of autochthonous cases of canine leishmaniasis in regions South of Brazil, which was considered non-endemic until 2008 (Porto Alegre, 2011). The State of Santa Catarina was considered free of VL until 2010, when autochthonous cases of canine visceral leishmaniasis were first diagnosed (Santa Catarina, 2020), underscoring the zoonotic significance of the disease.

This survey was a response to the occurrence of sandflies in neighboring States and countries to Santa Catarina, as well as records of sandfly presence in some regions of the State, particularly in eastern and coastal areas, coupled with an increase in canine leishmaniasis cases in the city.

During the assessment of the clinical and epidemiological records of the monitored dogs, it was clear that all animals either originated from or had moved to areas recognized as endemic for VL. These findings suggest that the animals were infected while traveling to other regions, possibly at their final destination or from their origin (Table 1), supported by this study’s finding of no sandflies in Lages. All vaccinated dogs received Leish-Tec® (Hertape Calier Saúde Animal), a vaccine that allows differentiation between naturally infected and vaccinated animals. The dogs were treated with Milteforan® and insect control measures, such as repellent collars, were implemented in accordance with Brazilian legislation. The dogs underwent mandatory monitoring every six months using conventional PCR to ensure adequate parasite control.

Lages currently lacks a mosquito control program, instead conducting periodic entomofauna surveys to monitor epidemiologically relevant vector species, including sandflies, none of which have been recorded to date. The absence of these species in the municipality may be attributed to its climatic conditions; however, this scenario could change in the near future due to current climate change trends and their impact in vector-borne diseases dynamics, potentially leading to the expansion of cases, including autochthonous ones, into previously free areas. At this time, the absence of sandflies in the municipality, combined with epidemiological factors related to dogs, supports the characterization of these cases as non-autochthonous and suggests that the area remains free from endemic transmission of the disease.

## Conclusion

This was the first comprehensive survey of the city’s entomofauna and the epidemiological analysis of documented canine leishmaniasis cases. Diptera was identified as the most prevalent order of insects in the municipality of Lages; however, no sandflies were detected. This absence may be attributed to the city’s climatic conditions, which likely do not support the reproduction and sustained presence of these species. The monitoring of vectors responsible for relevant vector-borne diseases should consider their dynamics throughout the year, taking into account variable edaphoclimatic conditions, especially in areas with well-defined seasons, as well as potential disturbances due to climate change. Future studies should evaluate how these variables influence insect occurrence and frequency in the municipality.

An analysis of confirmed and documented cases of canine leishmaniasis in the city, particularly considering epidemiological factors such as animal movement patterns and the absence of sandflies, supports the argument that these cases are non-autochthonous. With ongoing climate change projections and their potential impact on vector-borne diseases, continued surveillance of sandflies and other vectors is crucial within the One Health framework, to support strategic control and prevention efforts in both endemic and non-endemic regions.

## References

[B001] Alvar J, Vélez ID, Bern C, Herrero M, Desjeux P, Cano J (2012). Leishmaniasis worldwide and global estimates of its incidence. PLoS One.

[B002] Botelho PSM, Silveira S, Lara FM (1976). Flutuação populacional do Curuquerê do Algodoeiro (*Alabama argillacea*, Hueb.), em 4 municípios do Estado de São Paulo. An Soc Entomol Bras.

[B003] Brasil (2016). Nota técnica n° 11/2016/CPV/DFIP/SDA/GM/MAPA.

[B004] Cabaniel GS, Rada LT, Blanco JJG, Rodriguez-Morales AJ, Escalera JP (2005). Impacto de los eventos de El Niño Southern Oscillation (ENSO) sobre la leishmaniosis cutánea en Sucre, Venezuela, a través del uso de información satelital, 1994-2003. Rev Peru Med Exp Salud Publica.

[B005] Chowdhury R, Kumar V, Mondal D, Das ML, Das P, Dash AP (2016). Implication of vector characteristics of *Phlebotomus argentipes* in the kala-azar elimination programme in the Indian sub-continent. Pathog Glob Health.

[B006] Costa AT, Dias ES, Souza AGM, Silva FOL, Machado-Coelho GLL (2019). Ecology of phlebotomine sand flies in an area of leishmaniasis occurrence in the Xakriabá Indigenous Reserve, Minas Gerais, Brazil. Rev Soc Bras Med Trop.

[B007] Dantas-Torres F (2009). Canine leishmaniosis in South America. Parasit Vectors.

[B008] Dantas-Torres F (2024). Canine leishmaniasis in the Americas: etiology, distribution, and clinical and zoonotic importance. Parasit Vectors.

[B009] Dias ES, França-Silva JC, Silva JC, Monteiro EM, Paula KM, Gonçalves CM (2007). Flebotomíneos (Diptera: Psychodidae) de um foco de leishmaniose tegumentar no Estado de Minas Gerais. Rev Soc Bras Med Trop.

[B010] Forattini OP (1962). Entomologia médica..

[B011] Google Earth (2024). Google Earth.

[B012] IBGE (2022). Lages: território.

[B013] Jeronimo SMB, Oliveira RM, Mackay S, Costa RM, Sweet J, Nascimento ET (1994). An urban outbreak of visceral leishmaniasis in Natal, Brazil. Trans R Soc Trop Med Hyg.

[B014] Nieves E, Oraá L, Rondón Y, Sánchez M, Sánchez Y, Rujano M (2015). Distribution of vector sandflies leishmaniasis from an endemic area of Venezuela. J Trop Dis.

[B015] PAHO (2023). Manual de procedimientos para la vigilancia y el control de las leishmaniasis en la Región de las Américas..

[B016] Pandolfo C, Braga HJ, Silva VP, Massignam AM, Pereira ES, Thomé VMR (2002). Atlas climatológico do estado de Santa Catarina.

[B017] Porto Alegre (2011). Leishmaniose visceral no Rio Grande do Sul. Bol Epidemiol.

[B018] Rangel EF, Vilela ML (2008). *Lutzomyia longipalpis* (Diptera, Psychodidae, Phlebotominae) and urbanization of visceral leishmaniasis in Brazil. Cad Saude Publica.

[B019] Régnière J, Powell J, Bentz B, Nealis V (2012). Effects of temperature on development, survival and reproduction of insects: experimental design, data analysis and modeling. J Insect Physiol.

[B020] Ricken P, Hess AF, Borsoi GA (2018). Relações biométricas e ambientais no incremento diamétrico de *Araucaria angustifolia* no Planalto Serrano Catarinense. Cienc Florest.

[B021] Rutledge LC, Ellenwood DA (1975). Production of phlebotomine sandflies on the open forest floor in Panama: the species complement. Environ Entomol.

[B022] Santa Catarina (2020). Vigilância da Leishmaniose Visceral Canina (LVC).

[B023] Schäfer I, Müller E, Naucke TJ (2022). Ein update zur leishmaniose des hundes: diagnostik, therapie und monitoring. Tierarztl Prax Ausg K Klientiere Heimtiere.

[B024] Silva JAO, Silva FJ, Macedo LO, Santos CVB, Alves LC, Ramos RAN (2019). Sandflies in an endemic area for Visceral Leishmaniasis in Northeastern Brazil. Rev Bras Parasitol Vet.

[B025] Torres de Sousa RLT, Nunes MI, Freire SM (2019). Perfil epidemiológico de pacientes com leishmaniose visceral notificados em hospital de referência em Teresina - PI. Rev Interdiscip Estud Saúde.

[B026] Vieira VF, Dortzbach D (2017). Caracterização ambiental e delimitação geográfica dos Campos de Cima da Serra.

